# Rural–urban disparities in patient satisfaction with oral health care: a provincial survey

**DOI:** 10.1186/s12903-021-01613-0

**Published:** 2021-05-15

**Authors:** Abdalgader Alhozgi, Jocelyne S. Feine, Farzeen Tanwir, Richa Shrivastava, Chantal Galarneau, Elham Emami

**Affiliations:** 1 Faculty of Dentistry, McGill University, 2001 Avenue McGill College #500, Montreal, QC H3A 1G1 Canada; 2 Sri Aurobindo College of Dentistry, Sanwer Road, Indore, India; 3 Faculty of Dentistry, Université de Montréal, C.P. 6128, succursale centre-ville, Montreal, QC H3C 3J7 Canada; 4 Institut National de Santé Publique du Québec, 190 Boul Crémazie E, Montreal, QC H2P 1E2 Canada

**Keywords:** Satisfaction, Disparity, Urban, Rural, Oral Health Care

## Abstract

**Background:**

Identifying spatial variation in patient satisfaction is essential to improve the quality of care. Thus, the objective of this study was to investigate rural–urban disparities in patient satisfaction and to determine the factors that could influence satisfaction with oral health care.

**Methods:**

Data from 1788 parents/caregivers of children who participated in the Quebec Ministry of Health clinical study were subject to secondary analysis. The Perneger model of patient satisfaction was used as the conceptual framework for the study. Satisfaction with oral health care was measured using the WHO-sponsored International Collaborative Study of Oral Health Outcomes (ICS-II). Explanatory variables included predisposing factors and enabling resources. Statistical analyses included descriptive statistics, as well as bivariate and linear regression models.

**Results:**

Individuals with higher income, dental insurance coverage, having a family dentist, reporting ease in finding a dentist, and having access to a private dental clinic were more satisfied with oral health care (p < 0.001). There were statistically significant differences between rural and urban Quebec residents in their ratings of patient satisfaction on four items, including dental office location (p = 0.013), dental equipment (p = 0.016), cost of dental treatment (p < 0.001), and cleanliness of dental office (p = 0.004), with greater satisfaction for urban dwellers. The multiple linear regression model showed that major determinants of patient satisfaction were being born in Canada, income ≥ 40,000$ CAD, having a family dentist, and having visited the dentist in the last year for regular checkups. However, ethnicity, having difficulty finding a dentist, and being in need of dental treatment negatively influenced patient satisfaction with oral health care.

**Conclusions:**

These findings suggest that Quebec rural–urban disparity exists in patient satisfaction with care and that determinants of health influence this outcome. Intensive and powerful knowledge dissemination activities are needed to mobilize policymakers in implementing public health strategies to reduce this disparity.

## Background

The evaluation of health care quality has traditionally been based on objective measurements of regulation bodies, such as harm and clinical errors, with less attention to patients’ experiences, expectations and ratings of care delivery [1]. In 2010, the Institute of Medicine (IOM) published guidelines for the improvement of quality health care systems, with an emphasis on patient centred-care, equity and efficiency of care [2, 3]. Accordingly, in the last decades of evidence-based practice, patients have been involved in clinical decision making and evaluation of care, and patient satisfaction has been used as an indicator of quality of health care [4–6]. Patient satisfaction is defined by Pascoe as “a patient’s response to a significant aspect of her/his experience of health care services” [7]. Satisfying patients results in positive health care outcomes, including increased trust in health care providers and health care systems [8].

Patient satisfaction entails numerous dimensions and is related to several factors, such as socio-economic background, cultural values, environmental characteristics of health care settings, accessibility and availability of care, patients’ previous experiences with health care, the quality and effectiveness of the treatment, as well as health care providers’ attitudes, experiences and knowledge [8, 9]. Demographic and socio-economic characteristics of patients including age, gender, place of residency, education, income, marital status, and race are considered to be among the main determinants of patient satisfaction with health care [10, 11]. According to the Batbaatar et al. [11] systematic review, the determinants of patient satisfaction include the environment where the care is delivered, as well as the accessibility of care. Numerous studies have demonstrated that easy access to care increases patient satisfaction [7, 11–14]. In addition, research has indicated that patient satisfaction is strongly linked to the distance where the health care facilities are located, as well as waiting time [11, 15–26]. Furthermore, these studies showed that availability is dependent on available workforce and affordability, as well as flexibility of payment mechanisms, insurance status and insurance coverage; all of these increase patient ratings of satisfaction [11, 27].

Some studies showed that rural communities have lower levels of satisfaction with health care than their urban counterparts [28, 29]. Rural population are reported to have a poor quality of life, access to oral health, and oral health status [30–34]. People living in rural areas may also be less satisfied with their oral health care because of poor access to oral health care services that, in turn, could be influenced by several factors such as low socioeconomic status, geographic remoteness, shortages of dental professionals, lack of public transportation and limited dental insurance coverage [28, 35–38]. According to the Canadian Census 2016, about 19.4% of all Quebecers live in rural areas [39]. However, as per the study conducted by Emami et al., significantly higher number of general and specialists dentists (90.3%) are located in urban areas in Quebec [40]. Identifying spatial variation in patient satisfaction is essential to improve the provision of quality oral care [28]. According to available evidence, there is no study that examines rural–urban disparity in patient satisfaction with oral health care. Thus, additional research is needed to fill this knowledge gap. Therefore, the objective of this study was to investigate rural–urban disparities in patient satisfaction with oral health care and to determine factors that may influence patient satisfaction with oral health care.

## Methods

### Design, setting, study participants

This study used the data (n = 1788) from a previous survey entitled “Dent Ma Region” [34]. The research study methodology has previously been published [34]. In brief, this study was an *add-on* to a provincial survey on the oral health status of Quebec’s primary school students (second grade & sixth grade school children), carried out in collaboration with the Institut National de Santé Publique du Québec (INSPQ) and the Quebec Ministry of Health and Social Services (MSSS). The target population of this survey was parents/caregivers of school children (second grade & sixth grade) who were living in the eight regions of the province of Quebec (not included North of Quebec, Indigenous population). A random subsample of the provincial survey was selected for the *Dent Ma Region* study using stratified two-stage data sampling. Rurality was defined according to the 2006 Metropolitan Area and Census Agglomeration Influenced Zone (MIZ) provided by Statistics Canada [41]. The residential postcode was used to assign respondents’ census geography using the Postal Code Conversion File Plus (PCCF4F+) [42]. Over sampling of certain schools in some rural areas was done to increase the reliability and precision of estimates for these regions. The sampling design considered the proper weighting of each unit and unequal probabilities of each sample, to be representative of rural–urban population of parents/caregivers of school children. Parents/caregivers, who agreed to participate after learning about the study from the dental examiner or the hygienist responsible for the INSPQ, received questionnaire packages with informed consent forms sent from the administrators of their children’s schools. Ethical approval was provided by the institutional review boards of the Université de Montréal and McGill University.

### Study outcome and data collection

An adapted Perneger’s model of patient satisfaction was used as the conceptual framework to analyse the study results [43]. According to this model, patient satisfaction with health care is associated with the patient experience and the quality of care, as well as patient characteristics [43].

Patient satisfaction with care was measured using a validated and highly reliable instrument (α = 0.87) developed originally for the WHO-sponsored International Collaborative Study of Oral Health Outcomes (ICS-II) [44]. The questionnaire contains 12 items, with each item scored on a four-point Likert-type scale (very satisfied = 4, fairly satisfied = 3, dissatisfied = 2, very dissatisfied = 1). The total summary score was calculated by adding the scores for all items, resulting in a total summary score ranging from 12 to 48 [45].

Andersen’s behavioral model of health services utilization was used to examine to what extent predisposing, enabling factors and the need for care could influence patients’ satisfaction with oral health care [34, 46, 47]. Accordingly, the predisposing factors included age, gender, ethnicity (African, South American, Indigenous, European, North American, Asian, Middle Eastern), marital status, place of birth, language, education, occupation, perceived general and oral health, oral health knowledge and place of residency. Enabling resources included household income, dental insurance coverage, having a family dentist, difficulty in finding a dentist, distance to dental care provider in kilometers, means of transportation to dental care services and type of dental care providers. Questions about dental visits during the past year, the need for dental treatment and reason for dental visit were asked to evaluate the perceived dental care need. These data were collected through a self-administered and validated multi-dimensional questionnaire [34, 45]. The items in this questionnaire were extracted from the Canadian Oral Health Measures Survey and the Quebec Oral Health Surveillance questionnaire [34, 48, 49].

### Data analysis

The data were weighted prior to the data analyses and were adjusted to take into account the survey design (value of 1.5). The data were first subjected to descriptive statistical tests to determine frequency counts, percentages, and univariate means, as well as to test for normality. Student’s t-tests and Pearson’s correlations were used to examine the association of independent variables with patients’ satisfaction ratings. All independent variables were incorporated into the multiple linear regression model to determine which of these variables are associated with total mean scores of patient satisfaction. The statistical significance was set at *p* ≤ 0.05, and data analyses were carried out using SPSS software version 20.0 (SPSS Inc., Chicago, IL, USA). All methods were carried out in accordance with the STROBE guidelines for reporting a cross sectional study [50].

## Results

The data set contained 1788 participants. The sample population was representative of the Quebec rural–urban population with approximately 19.0% (n = 333) living in rural areas and 81.4% (n = 1455) living in urban areas. The mean age of the sample was 39.3 ± 5.2 years. Most of the survey respondents were women (87.5%), married (87.5%) and had college/university education (81.0%). Most of them were employed full time (70.1%), with an annual income of ≥ 40,000$ CAD (56.7%). Rural residents had lower education and employment rates, as well as lower incomes than their urban counterparts.

Bivariate analysis showed that the mean total patient satisfaction score for the sample was high (42.3 ± 4.5), and there was no significant difference in this score regarding place of residency (*p* = 0.66; Table 1). However, females, born in Canada, North Americans, married and those having oral health knowledge and good perceived oral and general health were more satisfied than their counterparts (*p* < 0.05). Respondents having incomes greater than ≥ 40,000$ CAD, dental insurance coverage, a family dentist, ease in finding a dentist and access to private dental clinics were more satisfied, as well (*p* < 0.001). Those who visited the dentist in the previous year, having no dental treatment needs and visited dentist for regular checkups rated their satisfaction higher with oral health care (*p* < 0.001; Table 1).

**Table 1:** Bivariate analysis of predisposing factors, enabling factors, dental treatment needs (Andersen’s behavioral model of health services use-based variables) associated with patient satisfaction

Satisfaction differences	Mean ± SD	T	Mean difference	Std. error	95% Confidence interval	*p* value
Gender						
Male	41.8 ± 4.6	− 2.04	− 0.663	0.32	(− 1.30, − 0.03)	0.042
Female	42.4 ± 4.5					
Place of birth						
Canada	42.6 ± 4.4	7.72	3.25	0.42	(2.43, 4.08)	< 0.001
Others	39.3 ± 4.4					
Ethnicity						
North American	42.5 ± 4.3	4.51	1.80	0.40	(1.02, 2.58)	< 0.001
Others	40.7 ± 5.6					
Marital status						
Single	41.5 ± 4.7	2.87	0.94	0.33	(0.30, 1.57)	0.004
Married	42.4 ± 4.5					
Income						
< 40,000$ CAD	41.7 ± 4.8	− 4.54	− 1.04	0.23	(− 1.49, − 0.59)	< 0.001
≥ 40,000$ CAD	42.8 ± 4.3					
Dental knowledge						
Yes	42.4 ± 4.5	3.08	1.93	0.63	(0.70, 3.16)	0.002
No	40.5 ± 4.7					
Perceived general health						
Poor	38.9 ± 4.4	4.53	3.51	0.77	(1.99, 5.02)	< 0.001
Good	42.4 ± 4.5					
Perceived oral health						
Poor	39.0 ± 5.1	7.03	3.50	0.50	(2.52, 4.47)	< 0.001
Good	42.5 ± 4.4					
Dental insurance						
Yes	42.8 ± 4.4	6.50	1.47	0.23	(1.03, 1.91)	< 0.001
No	41.4 ± 4.7					
Type of clinic						
Private	42.4 ± 4.5	3.22	1.64	0.51	(0.64, 2.64)	0.001
Public	40.8 ± 5.0					
Difficulty finding dentist						
Difficult	39.0 ± 5.1	− 8.04	− 3.53	0.44	(− 4.39, − 2.67)	< 0.001
Easy	42.6 ± 4.4					
Having family dentist						
Yes	42.6 ± 4.3	11.21	5.31	0.47	(4.39, 6.24)	< 0.001
No	37.3 ± 5.2					
Dental treatment need						
Yes	41.7 ± 4.7	− 4.78	− 1.07	0.22	(− 1.50, − 0.63)	< 0.001
No	42.7 ± 4.4					
Having dental visit last year						
Yes	42.7 ± 4.2	8.36	2.51	0.30	(1.29, 3.10)	< 0.001
No	40.2 ± 5.4					
Reason of visiting dentist						
Check up	42.9 ± 4.4	5.30	1.13	0.21	(0.71, 1.55)	< 0.001
Other reasons	41.8 ± 4.6					
Place of residency						
Rural	42.2 ± 4	0.43	0.12	0.28	(− 0.42, 0.66)	0.66
Urban	42.4 ± 4.6					

Regarding individual questionnaire items, all study participants highly rated their satisfaction with cleanliness and neatness of the dental office, whereas the lowest mean item score was for the cost of the last dental visit.

As shown in Table 2, there were statistically significant differences between rural and urban Quebec residents in patient ratings of four satisfaction items, including: dental office location (*p* = 0.013), dental equipment *(p* = 0.016), cost of dental treatment (*p* < 0.001) and cleanliness of the dental office (*p* = 0.004). Rural residents were less satisfied than their urban counterparts with the neighborhood where the dental office was located, as well as the cost of their last dental visit. In contrast, urban residents were less satisfied with the dental equipment and the cleanliness of the dental office than were rural residents.

**Table 2:** Rural–urban differences: patient satisfaction with Oral Health Care

Questionnaire’s item	Urban mean ± SD	Rural mean ± SD	Mean difference	Std. error	95% Confidence interval	*p* value*
Getting an appointment when you wanted it	3.3 ± 0.6	3.3 ± 0.7	− 0.002	.039	(− 0.78, 0.75)	0.70
The time it took to get there?	3.4 ± 0.7	3.4 ± 0.6	− 0.007	0.039	(− 0.084, 0.071)	0.87
The neighborhood where the dental office is located	3.6 ± 0.6	3.5 ± 0.5	0.085	0.034	(0.018, 0.152)	0.013
The way you were made to feel welcome by the receptionist?	3.7 ± 0.5	3.7 ± 0.5	0.025	0.029	(− 0.033, 0.083)	0.39
The way you were made to feel welcome by the hygienist/dental chairside assistant?	3.7 ± 0.5	3.7 ± 0.5	0.037	0.028	(− 0.092, 0.018)	0.19
The way you were made to feel welcome by the dentist?	3.7 ± 0.5	3.7 ± 0.5	0.029	0.028	(− 0.026, 0.084)	0.30
The information given you about what was wrong with your teeth?	3.6 ± 0.2	3.6 ± 0.5	0.010	0.038	(− 0.052, 0.072)	0.75
The information given you about what treatment was provided for you?	3.6 ± 0.5	3.7 ± 0.5	− 0.040	0.032	(− 0.102, 0.022)	0.21
How up to date the dental equipment is?	3.6 ± 0.5	3.7 ± 0.5	− 0.079	0.033	(− 0.142, − 0.015)	0.016
The cost of your last dental visit?	3.0 ± 0.8	2.8 ± 0.9	0.193	0.049	(0.097, 0.289)	< 0.001
The amount of time you waited to see the dentist?	3.4 ± 0.6	3.5 ± 0.6	0.193	0.049	(− 0.083, 0.061)	0.79
The cleanliness and neatness of dental office	3.7 ± 0.5	3.8 ± 0.4	− 0.077	0.027	(− 0.129, − 0.024)	0.004

^*^Adjusted for the effect plan

The multiple linear regression model showed that major determinants of patient satisfaction were being born in Canada (p < 0.001), income ≥ 40,000$ CAD (p < 0.05), having a family dentist (p < 0.001), and having visited the dentist in the last year for regular checkups (p < 0.05). However, ethnicity (p < 0.05), having difficulty finding a dentist (p < 0.001), and being in need of dental treatment (p < 0.05) negatively influenced patient satisfaction with oral health care (Table 3). These factors explained 17% of the variability in patient satisfaction ratings.

**Table 3:** Multiple linear regression model factors associated with patient satisfaction total scores

Variable	*B*	*SE B*	*β*	*t*	95% Confidence interval	Sig
Place of birth (ref: Canada)	2.86	0.69	0.16	4.11	(1.49, 4.22)	< 0.001
Ethnicity (ref: North American)	− 1.13	0.57	− 0.07	− 1.98	(− 2.25, − 0.01)	0.048
Income (ref: ≥ 40,000$)	0.68	0.31	0.07	2.14	(0.05, 1.30)	0.032
Difficulty to find a dentist (ref: Yes)	− 3.51	0.58	− 0.17	− 6.03	(− 4.65, − 2.36)	< 0.001
Need for dental treatment (ref: Yes)	− 0.70	0.29	− 0.07	− 2.44	(− 1.27, − 0.13)	0.015
Having family dentist (ref: Yes)	4.13	0.74	0.19	5.56	(2.67, 5.59)	< 0.001
Visiting the dentist in last year (ref: Yes)	0.87	0.40	0.06	2.14	(0.07, 1.66)	0.032

The multiple linear regression analysis included all the independent variables

## Discussion

Patient satisfaction is a complex and multi-factorial concept [11, 51–57]. Satisfaction with dental care services has been previously studied, since patients’ evaluation of the quality and experience of care is instrumental to the improvement of quality of services [57–60]. Nevertheless, disparities in these services remain a challenge for user access to health and oral health services globally, particularly for people who live in rural and remote neighborhoods [30, 38]. Spatial disparity that could influence patient satisfaction in oral health care has previously been highlighted in the literature [6, 38, 53, 61, 62]. To the best of our knowledge, this is the first study that investigates rural–urban disparities in patient satisfaction with oral health care in a Quebec population.

In our study, enabling resources and predisposing factors were associated with patient ratings of satisfaction with care. People living in rural areas were less satisfied with the location of the dental office and cost of dental treatment than those in urban settings. These results are in line with the findings of a recent systematic review [11] in which the determinants of patient’s satisfaction were shown to include patients’ characteristics, affordability of care and access to care [11]. There is overwhelming evidence that rural residents must travel long distances to access dental care and that they have less dental insurance coverage [6, 34, 38, 63]. Accordingly, our study highlighted that determinants of health, including social determinants, have an impact on patient satisfaction with care. As our study is in line with the Perneger [43] theoretical concept, factors such as expectations of health care, health care provider attitudes, quality of the health care and patient characteristics could all influence patient satisfaction [7, 43, 64]. According to previous dental literature [43, 45], meeting patients’ expectations produces greater satisfaction with care. In line with other study findings, our investigation showed that the facilities and cleanliness of a dental office could influence patient satisfaction; urban residents were less satisfied with dental equipment and cleanliness of the dental office [63, 65] than were rural residents. In fact, they might have higher expectations of dental care, as they have greater access to dental offices with high end technologies and facilities [6, 58, 66, 67].

We found no rural–urban differences in general satisfaction with care scores; this finding agrees with the results of previous studies that indicate patients are generally satisfied with oral health care [65, 67]. Geographical location was not the key determinant of satisfaction for rural population in our study. This could be because of an adaption to rural conditions, for example in terms of longer distance tolerance for doctor visits [68] and having fewer expectations regarding patient satisfaction with health care [69, 70]. High satisfaction ratings also could be attributed to the ceiling effect [71] or may suggest that individual item scoring might be a more sensitive measure for the quality and experience with care than global scoring. Respondents are generally reluctant to express negative opinions or openly disagree and try to give socially acceptable answers, possibly because of cultural differences in communication or attitude of patients [28, 37, 51].

Our regression model demonstrates that those who were born in Canada have higher satisfaction scores than those were not, possibly because the latter may face some kind of racial or ethnic discrimination that is not necessarily sensitive to their needs [11, 43, 72]. In our study, patients who visited the dentist for checkups showed higher satisfaction scores [67]. Regular checkups indicate increased compliance, fewer missed appointments, fewer pain episodes and a decreased need for advanced treatment [67, 73]. Not attending dental clinics regularly suggests a lack of awareness regarding the importance of oral health [34, 74]. Thus, improving public dental awareness in order to promote regular dental checkups by oral health professionals might increase patient satisfaction with dental care received [63, 67]. Evidence shows that rural–urban differences in patient satisfaction with health care vary by domain/aspect such as cultural factors, health care service provision and quality, expectations and utilization of healthcare services, physical facility, reliability, responsiveness, assurance and empathy [75–77]. In fact, rural residents in the US reported better satisfaction with staff responsiveness and care setting quietness than their urban peers, but lower satisfaction on cleanliness of a health care setting [78]. Similarly, an Indonesian study found that rural populations were more satisfied with responsiveness and less satisfied with empathy, whereas urban counterparts were the opposite. [75]. On the other hand, polish rural patients admitted to a large urban hospitals were more satisfied with hospital settings, staff care, doctors’ professional skills, and hospitalization outcomes [77]. Similarly, a Scottish study identified higher satisfaction in the rural populations with their local doctors and local healthcare resources [79]. In contrast, there was no significant difference in satisfaction with primary health care services between rural and urban Ghanaian women [28].

The results of our study should be interpreted with caution due to certain limitations. Firstly, our study results cannot be generalized to other geographical areas, as this study was conducted with Quebec residents. Secondly, the questionnaire used to measure patient satisfaction didn’t include items regarding provider-level quality of care and interpersonal experience between providers and patients. Lastly, since the study was an *add-on* to the MSSS provincial clinical study, there were some limitations regarding narrow variability in age and gender. On the other hand, the strength of our study lies in the large sample size, and the dimensions used in our survey could possibly be used in future studies in the assessment of dental care.

## Conclusion

These findings suggest that Quebec rural–urban disparities exist in patient satisfaction with care and that the determinants of health influence this outcome. Intensive and powerful knowledge dissemination activities will help to mobilize policy makers in implementing public health strategies to reduce this disparity.

## Data Availability

The datasets used and/or analysed during the current study are available from the corresponding author on reasonable request.
